# Cytotoxicity and Radiosensitizing Activity of the Fatty Acid Synthase Inhibitor C75 Is Enhanced by Blocking Fatty Acid Uptake in Prostate Cancer Cells

**DOI:** 10.1016/j.adro.2020.06.022

**Published:** 2020-06-29

**Authors:** Colin Rae, Georgios I. Fragkoulis, Anthony J. Chalmers

**Affiliations:** Wolfson Wohl Translational Cancer Research Centre, Institute of Cancer Sciences, University of Glasgow, Glasgow, Scotland, United Kingdom

## Abstract

Prostate cancers, like many other types of cancer, express elevated levels of fatty acid synthase (FASN) to make more fatty acids, which are required for energy, signaling, and proliferation. Because inhibition of FASN has been shown to sensitize tumors to chemotherapy and radiation, we studied the effect of C75, a radiosensitizing FASN inhibitor, and compared its single agent and radiosensitizing activities in 2 prostate cancer cell lines, PC3 and LNCaP, with alternative FASN inhibitors that have progressed into clinical trials. We also investigated the effect of serum and fatty acid supplementation on responses to FASN inhibitors, probing expression of key proteins related to fatty acid uptake in response to FASN inhibition, irradiation, and serum lipid concentration and how this may be modulated to increase the potency of C75. We demonstrated that C75 was the only FASN inhibitor to sensitize cells to ionizing radiation; no sensitization was apparent with FASN inhibitors TVB-3166 or Orlistat. The prostate cancer cell lines were able to take up fatty acids from the culture medium, and the availability of fatty acids affected sensitivity of these cells to C75 but not the other FASN inhibitors tested. C75 also increased expression of fatty acid transporter proteins FATP1 and CD36. Furthermore, blocking CD36 with antibody increased the sensitivity of cells to C75. We suggest that the potency of C75 is affected by fatty acid availability and that the effectiveness of FASN inhibitors in combination with ionizing radiation can be further enhanced by regulating fatty acid uptake.

## Introduction

Prostate cancer is the most commonly diagnosed malignancy in men and the second leading cause of cancer-related deaths in men in industrialized countries. Many common human cancers, including prostate carcinoma, have elevated levels of lipogenesis and express high levels of enzymes associated with fatty acid synthesis compared with normal human tissues.^1^^,^^2^ This increased lipogenesis, possibly regulated by androgens, is an early event in the development of prostate cancer^3^ and correlates with unfavorable prognosis and poor survival. The enzyme responsible for endogenous synthesis of saturated long-chain fatty acids from the precursors acetyl-CoA and malonyl-CoA is fatty acid synthase (FASN). Most human tissues, except liver and adipose tissue, preferentially use circulating dietary fatty acids, and therefore FASN is expressed at low levels in these tissues. However, elevated expression of this enzyme in tumors is associated with proliferation, resistance to apoptosis, and increased metastasis.^4^

Inhibition of FASN has been shown to decrease cancer cell proliferation, increase apoptosis, and delay tumor growth in experimental models.^5^, ^6^, ^7^ Cerulenin is a FASN inhibitor that is cytotoxic to cancer cells in vitro but lacks systemic activity in vivo.^8^ Synthetic derivatives of cerulenin, such as the competitive irreversible FASN inhibitor C75 (α-methylene-β-butyrolactone), have been developed, and they display anti-tumor activity in preclinical models.^9^ However, progress to the clinic of FASN inhibitor drugs has been hampered by poor pharmacokinetics and associated side effects, particularly weight loss and anorexia, which limit their potential for the treatment of patients with cancer.^10^^,^^11^

Radiation therapy is an effective and well-tolerated treatment option for many patients with prostate cancer. However, resistance is common and may be overcome by combining radiation therapy with radiosensitizing agents. Similar to the chemosensitization by FASN inhibitors when administered in combination with anti-cancer drugs,^12^^,^^13^ previous studies have demonstrated that FASN inhibition can sensitize cancer cells to radiation therapy in experimental models.^14^, ^15^, ^16^ However, there are currently no FASN inhibitors that are clinically approved for use in cancer therapy and the radiosensitizing ability of FASN inhibitors in clinical trials has not been fully evaluated.

Although Orlistat is a FASN inhibitor that is approved only as an anti-obesity drug, it has also been shown to affect cancer cells. It decreased proliferation and induced apoptosis in prostate cancer cell lines PC3 and LNCaP and inhibited growth of PC3 and LNCaP xenograft tumors without affecting proliferation and survival of non-tumor cells.^16^^,^^17^ Orlistat has also been shown to inhibit angiogenesis and metastasis in preclinical models^18^^,^^19^ and appears to sensitize prostate cancer cells to radiation therapy in a xenograft model of prostate cancer.^16^

Saginet Biosciences (formerly 3-V Biosciences) (TVB) has developed a series of imidazopyridine compounds^20^ that selectively inhibit FASN and that demonstrated anti-tumor activity in preclinical models. One of these compounds, TVB-2640, has become the first FASN inhibitor to enter clinical trials for patients with cancer, and initial reports have demonstrated encouraging responses in patients with non-small cell lung, ovarian, and breast cancer.^21^ TVB-3166 and its analogs, TVB-3644 and TVB-3693, decreased proliferation, increased apoptosis, regulated signaling pathways associated with proliferation and survival, and decreased growth in multiple tumor cell lines and patient-derived xenografts.^22^, ^23^, ^24^ In studies using 22Rv1 prostate cancer cells, TVB-3166 induced apoptosis, reduced growth, and enhanced the anti-tumor effect of taxanes in a xenograft model.^24^^,^^25^ Therefore, we intended to assess the ability of this FASN inhibitor to sensitize prostate cancer cells to radiation in our models.

In an attempt to overcome the side effects associated with C75, enantiomers of this drug have been developed. It was shown that the (-)-C75 enantiomer was responsible for the anti-tumor properties, whereas (+)-C75 induced the anorectic effects associated with the commonly used racemic mixture, (±)-C75.^26^ Cytotoxicity of the (-)-C75 enantiomer was also similar to (±)-C75 in PC3 and LNCaP, and importantly, the radiosensitizing properties of (-)-C75 were similar to those of (±)-C75,^27^ indicating that it may be possible to retain the anti-tumor and radiosensitizing effects of this drug while preventing the unwanted weight loss. This highlights the importance of understanding the mechanisms by which C75 induces radiosensitization and whether other FASN inhibitors have similar effects when directly compared in the same models.

In addition to increasing the specificity of FASN inhibitors for clinical application, mechanisms by which cancer cells are resistant to these agents should also be investigated. It is becoming evident that uptake of exogenous lipids from the tumor microenvironment plays an important role in disease progression and resistance to treatment. Indeed, it has recently been demonstrated that fatty acid uptake is increased in prostate cancer cells compared with non-tumor tissue,^28^ and expression of fatty acid transporters is regulated by androgens.^29^ Accordingly, the therapeutic potential of targeting both lipid uptake and lipogenesis has been proposed. Therefore, we sought to compare the cytotoxicity and radiosensitizing ability of FASN inhibitors and investigate whether this was affected by exogenous fatty acid availability and the expression of fatty acid transporters.

## Methods and Materials

### Reagents

All cell culture media and supplements were purchased from Life Technologies (UK) unless stated otherwise. All chemicals, drugs, and oleic acid-bovine serum albumin solution were from Sigma-Aldrich (UK). Stock solutions of drugs were prepared in dimethyl sulfoxide (DMSO). Vehicle control treatments contained DMSO in culture medium. For antibody treatments, 10 μg/mL CD36 blocking antibody (mouse monoclonal; Santa Cruz Biotechnology, Germany) or control antibody (mouse IgG isotype control; Insight Biotechnology, UK) was used.

### Tissue culture

Human prostate cancer cell lines, PC3 and LNCaP, were obtained from American Type Culture Collection (Manassas, VA) and were used in this study for less than 6 months after resuscitation. PC3 cells were maintained in Ham’s F-12K medium supplemented with 10% (v/v) fetal bovine serum (Autogen Bioclear, Wiltshire, United Kingdom), 2 mM L-glutamine, 0.1 mM sodium pyruvate, and 50 μg/mL gentamicin. LNCaP cells were maintained in RPMI 1640 medium supplemented with 10% (v/v) fetal bovine serum (Hyclone; Fisher Scientific, United Kingdom), 1% (v/v) hydroxyethyl piperazineethanesulfonic acid (HEPES buffer), 1% (v/v) D-glucose, 1 mM sodium pyruvate, 4 mM L-glutamine, and 50 μg/mL gentamicin.

For comparison, human neuroblastoma SK-N-BE(2c) and MCF7 cells were purchased from the American Type Culture Collection, and UVW glioma cells were obtained from the CRC Beatson Laboratories Medical Oncology Department in Glasgow, United Kingdom.^30^ SK-N-BE(2c) cells were maintained in Dulbecco’s modified Eagle medium containing 15% (v/v) serum and 2 mM L-glutamine; MCF7 in F12K medium containing 10% (v/v), 2 mM L-glutamine, 0.1 mM sodium pyruvate, and 50 μg/mL gentamicin; and UVW in minimal essential medium containing 10% (v/v) serum and 2 mM L-glutamine.

### MTT cytotoxicity assay

3-(4,5-dimethylthiazol-2-yl)-2,5-diphenyltetrazolium bromide (MTT) reduction assay was used to determine cell viability. LNCaP and PC3 cells were seeded in 96-well plates and incubated at 37^o^C, 5% CO_2_ to allow exponential growth. Medium was then replaced by fresh medium containing drugs, oleic acid, or antibodies at the required concentrations in triplicate wells. MTT was added to a final concentration of 0.5 mg/mL and incubated for 2 hours. Cells were then solubilized by addition of DMSO and the absorbance read at 570 nm using a Tecan M2000 plate reader.

### Clonogenic assay

Cells were seeded in 25 cm^2^ flasks at 10^5^ cells/flask. When cultures were in exponential growth phase, medium was removed and replaced with fresh medium (including 1% or 10% serum) containing drug. For radiation treatment, cells were irradiated using an X-Strahl RS225 x-ray irradiator (Xstrahl Limited, Surrey, United Kingdom) at a dose rate of 1.6 Gy per minute. Cells were then incubated for 24 h at 37ºC in 5% CO_2_. After treatment, cells were counted and seeded for clonogenic survival assay as previously described.^15^ Cells were incubated at 37°C in 5% CO_2_ for up to 13 days. LNCaP cells did not form clonogens under similar conditions and so were not used for clonogenic assay. Colonies were fixed in methanol, stained with crystal violet solution, and colonies of at least 50 cells were counted.

### Immunoblotting

PC3 and LNCaP cells were cultured in monolayers in 25 cm^2^ flasks for 3 days. Cells were then treated for 24 h before protein was extracted in lysis buffer containing protease and phosphatase inhibitors. Whole cellular protein extracts were resolved in reducing and denaturing conditions by sodium dodecyl sulfate polyacrylamide gel electrophoresis. Proteins were transferred onto polyvinylidene difluoride Immobilon-P membranes (Merck Millipore, United Kingdom). Membranes were blocked with 7.5% (w/v) nonfat dried milk before incubation with the primary antibodies overnight at 4°C. Antibodies against β-actin (Ab8224) and FATP1 (Ab69458) were obtained from Abcam, United Kingdom. Antibody against CD36 (sc-7309) was obtained from Santa Cruz Biotechnology. Membranes were then washed and incubated at room temperature for 1 hour with horseradish peroxidase-conjugated secondary anti-mouse antibody (Cell Signaling Technology, United Kingdom) to enable chemiluminescent detection using enhanced chemiluminescence (ThermoFisher Scientific, United Kingdom), and images were obtained using Image Lab software.

### Fatty acid uptake

Cells were seeded at a density of 1000 cells per well in 96-well plates and incubated in 37⁰C, 5% CO_2_ for 3 days. Medium was removed and replaced with oleic acid bound to bovine serum albumin in phosphate buffered saline (Sigma-Aldrich) for up to 72 hours. MTT absorbance was measured every 24 hours. Accumulation of lipids after 24 h was determined by Oil Red O staining. Briefly, medium was removed and cells washed with phosphate buffered saline before fixing with 10% paraformaldehyde. The cells were pre-treated with 60% isopropanol and then stained with 0.2% (w/v) Oil Red O (Sigma-Aldrich) in 60% isopropanol. Cells were then washed and photographed under the microscope. To solubilize, 100% isopropanol was added before reading absorbance at 492 nm.

### Statistics

The number of experimental repeats is provided in figure legends, and the data are presented in graphs as mean ± standard error of the mean. Where appropriate, results were analyzed using GraphPad Prism statistical software using Student *t* test or 1-way analysis of variance. A *P* value of ≤.05 was considered significant and ≤.01 highly significant.

## Results

### FASN inhibitors are cytotoxic to prostate cancer cell lines

**Figure 1:**
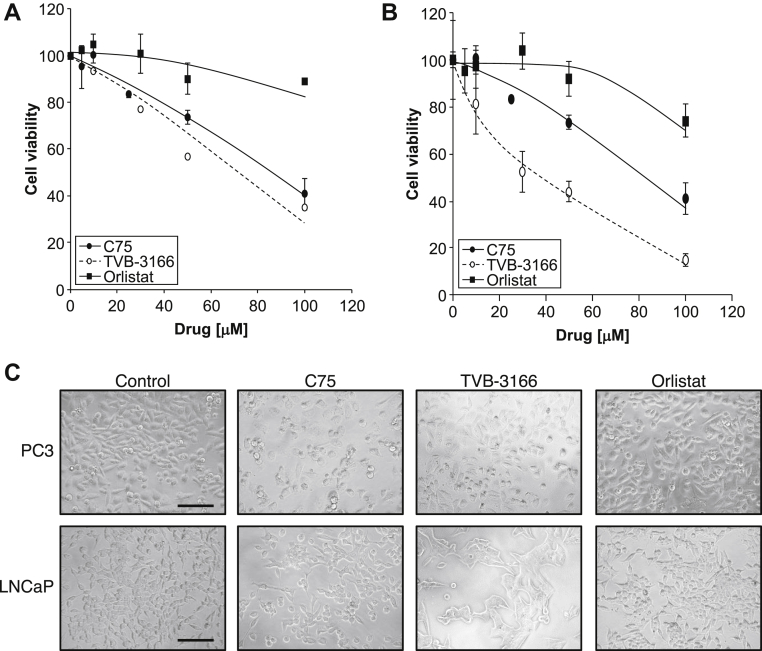
Cytotoxic effect of inhibitors of fatty acid synthase. Reduction of 3-(4,5-dimethylthiazol-2-yl)-2,5-diphenyltetrazolium bromide assay of (A) PC3 cells and (B) LNCaP cells after 24-hour exposure to C75, TVB-3166, or Orlistat. Data are means ± standard error of the mean (SEM), n = 3. (C) Representative images of PC3 and LNCaP cells after 24-hour treatment with control dimethyl sulfoxide (DMSO) or fatty acid synthase (FASN) inhibitors (50 μM drug) in medium containing 10% serum. Bars represent 200 μm.

The effect of 3 FASN inhibitors on viability of 2 prostate cancer cell lines, PC3 and LNCaP cells, was assessed using the MTT assay. When measured 24 hours after administration, cell viability was decreased in a concentration-dependent manner by all 3 FASN inhibitors (C75, TVB-3166, and Orlistat) in both PC3 and LNCaP cells, with Orlistat being the least potent (Fig 1A, B). The drugs induced distinct effects on cell morphology, with Orlistat and TVB-3166 causing cells (especially LNCaP) to become flattened, larger, and more granular (Fig 1C). In contrast, C75 treatment caused cells to round up and develop processes.

### Radiosensitizing effect of FASN inhibitors

**Figure 2:**
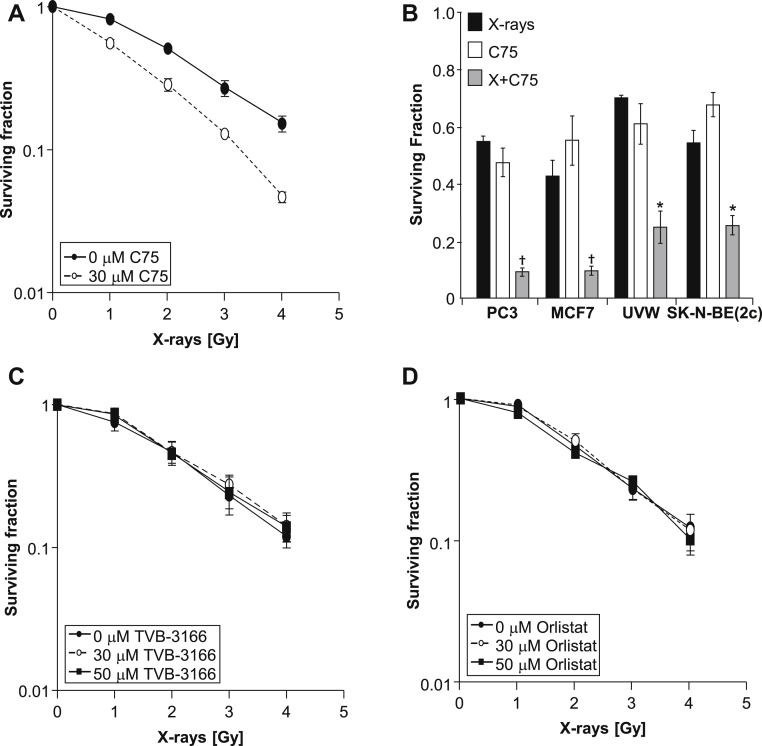
Combination of drugs with ionising radiation. (A) Clonogenic assay of PC3 cells 24 hours after simultaneous administration of x-rays and C75 (30 μM). (B) Clonogenic assay of PC3 (prostate cancer), MCF7 (breast cancer), UVW (glioma), and SK-N-BE(2c) (neuroblastoma) cells 24 hours after simultaneous administration of x-rays (2 Gy) and C75 (35 μM). Data are means ± standard error of the mean (SEM), n = 3. ^∗^*P* < .05 and ^†^*P* < .01 compared with single agent treatments. Clonogenic assay of PC3 cells 24 h after simultaneous administration of x-rays and (C) TVB-3166 or (D) Orlistat.

The effect of C75 on the radiation survival curve of PC3 prostate cancer cells is shown in Figure 2A, and the dose enhancement ratio at 50% clonogenic kill was calculated as 1.84 ± 0.21. These observations indicate radiosensitization by C75. The radiation-enhancing activity of C75 was also demonstrated in breast cancer (MCF7), glioma (UVW), and neuroblastoma cell lines (SK-N-BE[2c]) (Fig 2B) by showing supra-additive clonogenic kill after simultaneous administration of x-rays and C75. To evaluate the ability of alternative FASN inhibitors to sensitize prostate cancer cells to radiation, clonogenic assays were carried out on PC3 cells 24 hours after simultaneous administration of x-rays and TVB-3166 (Fig 2C) or Orlistat (Fig 2D). Neither TVB-3166 nor Orlistat (at concentrations of 30 and 50 μM) significantly enhanced the radiation-induced decrease in clonogenic survival, suggesting they were not acting in a similar manner to C75.

### Fatty acid availability affects sensitivity to C75

**Figure 3:**
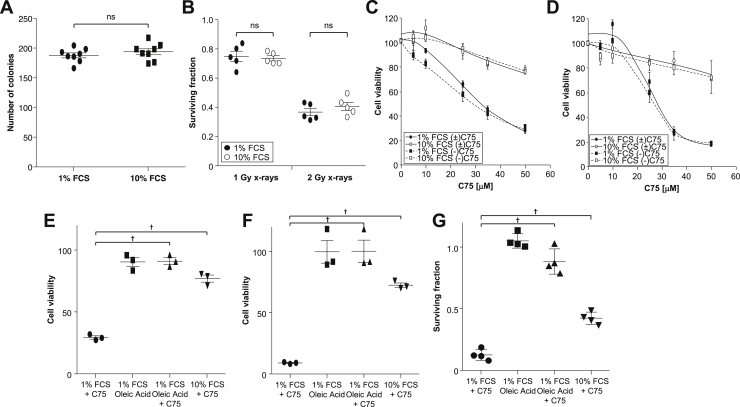
Serum concentration affects sensitivity to C75. (A) Clonogenic assay of PC3 cells after 24-hour culture in serum containing low (1%) or normal (10%) serum. (B) Surviving fraction of PC3 cells in clonogenic assay carried out 24 hours after exposure to x-rays (1 or 2 Gy). No significant difference was observed between treatments in 1% or 10% serum. 3-(4,5-dimethylthiazol-2-yl)-2,5-diphenyltetrazolium bromide assay of (C) PC3 cells and (D) LNCaP cells 24 hours after treatment with C75 administered as either the racemic mixture (±) or the (-) enantiomer in medium containing 1% or 10% serum. 3-(4,5-dimethylthiazol-2-yl)-2,5-diphenyltetrazolium bromide assay of (E) PC3 and (F) LNCaP cells was also carried out in 1% serum-containing medium with the addition of oleic acid-bovine serum albumin solution (100 μM oleic acid) and compared with the effect of C75 (50 μM) in 10% serum-containing medium. (G) Clonogenic assay of PC3 cells 24 h after culture in medium containing 1% or 10% serum or 1% serum with oleic acid (100 μM) with and without C75 (35 μM). Data are expressed compared with vehicle treated controls and are means ± standard error of the mean (SEM), n = 3, except (G) n = 4. ^∗^*P* < .05 and ^†^*P* < .01 compared with C75 in 1% serum-containing medium.

Because serum is a source of fatty acids, we further assessed differences in the response of prostate cancer cells to FASN inhibitors by carrying out experiments in reduced serum (1%) medium, thus reducing the availability of fatty acid. Compared with experiments carried out in 10% serum-containing medium, the number of clonogens was not affected by culture in 1% serum-containing medium for 24 hours before replating for clonogenic assay (Fig 3A). Similarly, alterations in serum level did not significantly affect the clonogenic kill of PC3 cells induced by administration of x-rays (Fig 3B).

In contrast, MTT assays demonstrated that alteration of serum levels significantly affected activity of both the racemic mixture of C75 (±) and the (-)-C75 enantiomer in both PC3 and LNCaP cells (Fig 3C, D), with cytotoxicity being more pronounced in cells cultured in medium containing 1% serum. As (-)-C75 had almost identical effects to (±)-C75, the racemic mixture was used in further experiments. Although addition of oleic acid to 1% serum medium had no direct effect on cell viability, it was sufficient to prevent C75-induced cytotoxicity in both PC3 and LNCaP cells (Fig 3E, F) to the level observed when cells were exposed to C75 in 10% serum medium. These effects were confirmed by clonogenic assays in PC3 cells (Fig 3G).

**Figure 4:**
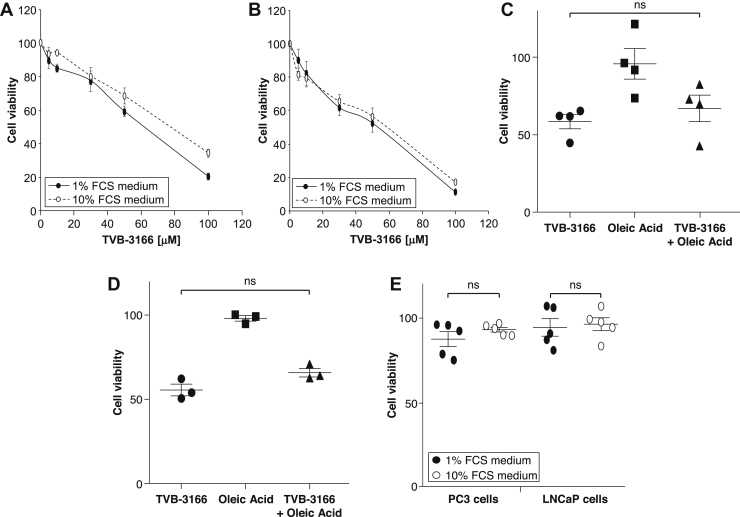
Serum concentration does not affect sensitivity to TVB-3166 or Orlistat. 3-(4,5-dimethylthiazol-2-yl)-2,5-diphenyltetrazolium bromide (MTT) assay of (A) PC3 and (B) LNCaP cells 24 h after administration of TVB-3166 in either 1% or 10% serum medium. (C) PC3 or (D) LNCaP cells were cultured in 1% or 10% serum medium with TVB-3166 (50 μM) and oleic acid (100 μM) before MTT assay. (E) MTT assay of PC3 and LNCaP cells 24 h after administration of Orlistat in 1% or 10% serum medium. Data are expressed compared with vehicle treated controls and are means ± standard error of the mean (SEM), n = 3-5.

In contrast to the effects on C75 activity, culture in low (1%) serum-containing medium led to only a small increase in TVB-3166-induced cytotoxicity in either PC3 or LNCaP cells (Fig 4A, B). Also, addition of oleic acid did not prevent the cytotoxicity induced by TVB-3166 in 1% serum medium (Fig 4C, D). Orlistat at a concentration of 50 μM had little cytotoxic effect on either PC3 or LNCaP cells, and this was not affected by reducing serum concentration from 10% to 1% (Fig 4E).

### Fatty acid uptake is regulated by availability

**Figure 5:**
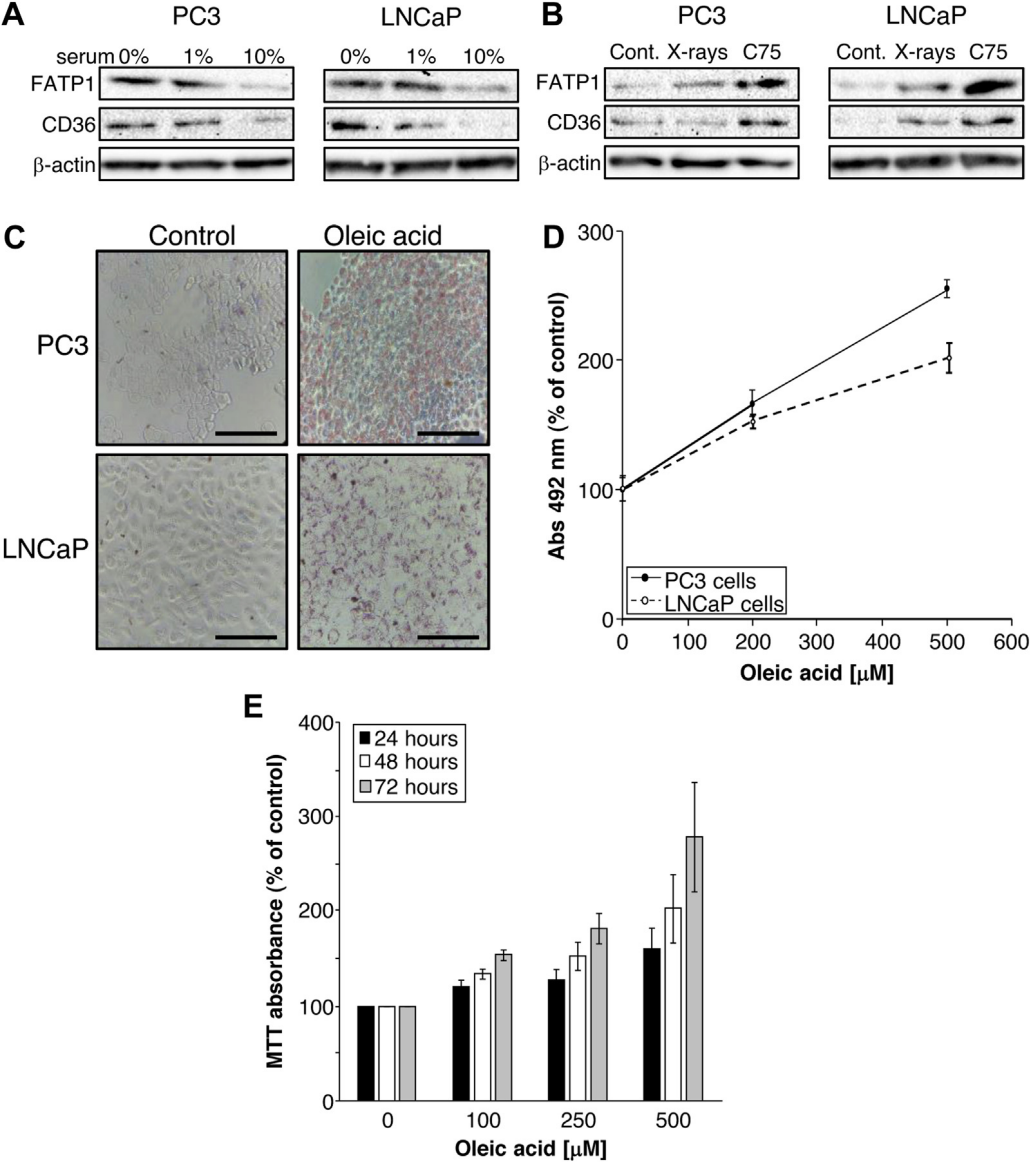
Lipid accumulation and fatty acid uptake in cells. (A) Protein expression of fatty acid transporter 1 (FATP1) and CD36 in PC3 and LNCaP cells was decreased by culture for 24 h in increased concentrations of serum in medium. (B) FATP1 and CD36 expression was increased by x-rays (2 Gy) and C75 (35 μM) in PC3 and LNCaP cells cultured in medium containing 10% serum. Representative blots are shown. Loading control was β-actin. (C) Representative images of lipid droplets in PC3 and LNCaP cells visualized by Oil Red O staining 24 h after culture in medium containing 1% serum with or without oleic acid (100 μM). Bars represent 500 μm. (D) Absorbance of PC3 cells and LNCaP cells exposed to oleic acid for 24 h then stained with Oil Red O. Stain was solubilized with isopropanol. n = 3. (E) 3-(4,5-dimethylthiazol-2-yl)-2,5-diphenyltetrazolium bromide assay of PC3 cells every 24 h after culture in medium containing 1% serum and 100 to 500 μM oleic acid. Data are expressed compared with vehicle treated controls and are means ± standard error of the mean (SEM), n = 4.

To further investigate whether the serum-dependent effects on cells and sensitivity to C75 were caused by differences in the fatty acid availability, we investigated the expression of proteins known to transport fatty acids into cells, fatty acid transporter 1 (FATP1), and CD36. Expression of both transporters was observed in PC3 and LNCaP cells cultured in serum-free (0%) or low (1%) serum, and decreased when cultured in normal (10%) serum (Fig 5A). Expression was also affected by treatment, with C75 increasing FATP1 and CD36 in both PC3 and LNCaP cells and x-rays inducing less marked upregulation (Fig 5B). Cells were able to respond to alterations in availability of fatty acids in medium, with Oil Red O staining indicating that both cell lines were able to take up and store fatty acids when the medium was supplemented with oleic acid (Fig 5C); this occurred in a concentration-dependent manner (Fig 5D). Furthermore, cells responded to increasing fatty acid availability by increasing proliferation (Fig 5E).

### Blockage of CD36 enhances cytotoxicity of C75

**Figure 6:**
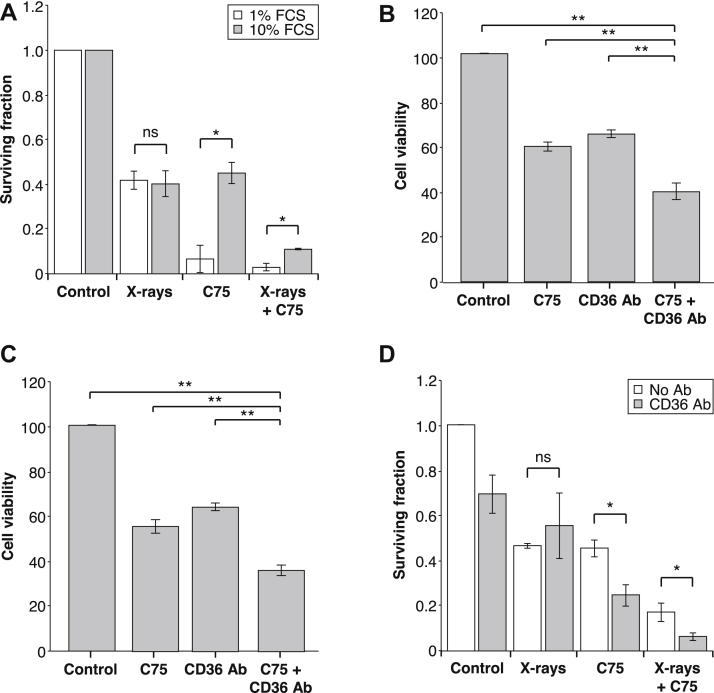
CD36 blocking antibody enhanced effect of C75. (A) Clonogenic assay of cells cultured for 24 h in medium containing 1% or 10% serum and treated with x-rays (2 Gy), C75 (35 μM), or a combination of both. ^∗^*P* < .05 between different serum concentrations. 3-(4,5-dimethylthiazol-2-yl)-2,5-diphenyltetrazolium bromide assay of (B) PC3 and (C) LNCaP cells in 10% serum medium after 24 h treatment with C75 (50 μM) in the absence or presence of CD36 antibody (10 μg/mL). (D) Clonogenic assay of PC3 cells treated in 10% serum containing medium for 24 h with x-rays (2 Gy), C75 (35 μM), or combination treatment in the absence or presence of CD36 antibody (10 μg/mL). Data are expressed compared with vehicle treated controls and are means ± standard error of the mean (SEM), n = 3. ^∗^*P* < .05 between presence and absence of antibody.

Although the clonogenic killing effect of x-rays was not significantly affected by serum concentration (Figs 3B and 6A), the radiosensitizing effect of C75 was enhanced in low-serum medium (Fig 6A). To confirm our hypothesis that this was caused by reduced availability and uptake of exogenous fatty acid, we investigated whether simultaneous administration of a CD36 neutralizing antibody would enhance the cytotoxic and radiosensitizing effect of C75. Neutralizing CD36 antibody decreased the viability of PC3 cultured in 10% serum medium and LNCaP cells when administered as a single treatment and enhanced the cytotoxicity of C75 in an approximately additive manner (Fig 6B, C). Control antibody (mouse IgG isotype control) was not cytotoxic (data not shown). Clonogenic assays in PC3 cells confirmed the potentiating effect of the CD36 antibody (Fig 6D).

## Discussion

Despite our previous demonstration that C75 radiosensitizes prostate cancer cells to radiation,^15^ concerns remain regarding the clinical application of this drug due to its unwanted effect on weight loss.^10^ Although several FASN inhibitors have been developed that display anti-cancer properties, it was important to determine whether more clinically applicable drugs share properties of C75 when directly compared using the same concentrations in the same models. First, we investigated the cytotoxic potency of 3 inhibitors of FASN, the cerulenin derivative C75, the anti-obesity drug Orlistat, and TVB-3166, one of a series of structurally related FASN inhibitors that includes TVB-2640, the first-in-human FASN for cancer clinical trials.^21^ Phase 1 clinical trials have also recently demonstrated the safe use of TVB-2640 in men with metabolic abnormalities.^31^ Orlistat was the least potent, with significant cytotoxicity observed in both PC3 and LNCaP cells around 100 μM, similar to previously reported observations in these and other cell lines.^14^^,^^16^ The cytotoxicity of TVB-3166 observed here was similar to C75 and was previously described at similar concentrations in bladder cancer cell lines.^24^ Inhibitors of FASN differ in their interaction with the enzyme, with Orlistat inhibiting the thioesterase domain of fatty acid synthase,^17^ whereas C75 binds to the β-ketoacyl synthase domain.^8^ These distinct effects may be exemplified by differences in the morphology of the cells after treatment observed here and previously described for C75 and Orlistat in LNCaP cells.^32^

Next, we sought to assess whether the other FASN inhibitors share with C75 the ability to enhance the radiation-induced kill of cancer cells. We have previously described the radiosensitizing effect of C75 in PC3 and LNCaP cells^15^ and now demonstrate this in cancer cell lines originating from breast cancer, glioma, and neuroblastoma. However, neither TVB-3166 nor Orlistat caused any enhancement of the radiation-induced clonogenic kill. Orlistat has previously been shown to sensitize only radioresistant (surviving cells from previous radiation treatment), but not radiosensitive, head and neck squamous cell carcinoma cells.^14^ Although Orlistat has recently been shown to sensitize PC3 and LNCaP cells to x-rays, this was in a xenograft model, where Orlistat enhanced the radiation-induced suppression of tumor growth.^16^ However, the in vivo effects were only observed at relatively high doses (240 mg/kg/d), and the clinical application of Orlistat for cancer therapy is potentially limited by poor solubility and low bioavailability.^33^ In comparable in vitro experiments, Chuang et al^16^ demonstrated similar levels of Orlistat cytotoxicity and x-ray-induced clonogenic kill in PC3 cells to those described here and previously,^15^ but the effect of the combination of Orlistat and x-rays on clonogenic kill was not reported. FASN inhibitors from TVB appear to be safe in humans and reduce lipogenesis in small clinical studies as a single agent, in addition to demonstrating some efficacy in patients with cancer.^21^^,^^31^ Although TVB-3166 has been shown to sensitize cancer cells to chemotherapy,^24^^,^^25^ the radiosensitizing efficacy of TVB-3166 has not previously been evaluated, and it appears from the current study that this FASN inhibitor does not radiosensitize in a similar manner to C75.

Inhibition of FASN may increase dependence on other components of fatty acid metabolism, including uptake of fatty acids.^34^ Therefore, we attempted to enhance the potency of C75 by modulating availability of exogenous fatty acids. Culturing in low-serum medium did not affect cell growth, clonogenic survival, or sensitivity to ionizing radiation, but markedly enhanced the potency of C75. Importantly, enantiomers of C75, developed to overcome the unwanted effects of racemic C75, displayed a similar serum dependence. Although the tolerability of (-)-C75 has already been reported in vivo,^26^ the anti-tumor effect and radiosensitizing effect have yet to be assessed in animal models. Reduced serum also increased sensitivity to TVB-3166, as previously described in colorectal cancer cells,^22^ although this was not as pronounced as with C75.

Monounsaturated fatty acids, such as oleic acid, may promote proliferation, migration, and invasion of cancer cells.^35^^,^^36^ Therefore, we evaluated the effect of culturing cells in medium containing additional oleic acid. As expected, we observed that C75-induced cytotoxicity was rescued by addition of oleic acid. However, the cytotoxicity induced by TVB-3166 was not significantly affected by oleic acid supplementation. Differences between C75 and TVB-3166 may reflect modulation of different intracellular signalling pathways. C75 increases activation of the metabolic sensing AMP-activated kinase pathway,^37^ which may also regulate sensitivity to radiation,^38^ whereas TVB-3166 affects signalling by disrupting the lipid raft architecture.^22^ In agreement with our findings with C75, decreasing monosaturated fatty acid availability by inhibition of the enzyme SCD1 decreased proliferation of breast cancer cells, and this effect was enhanced in low-serum condition and rescued by supplementation with oleic acid.^39^ Similarly, we showed the proliferative capacity of PC3 cells was increased in a concentration- and time-dependent manner when fatty acid availability was increased by addition of oleic acid to the medium, as also reported for these cell lines and others when medium is supplemented with oleic acid or a mixture of oleic acid and palmitic acid.^28^^,^^36^

Uptake of exogenous fatty acids is regulated by alterations in the expression of the FATP family, which contributes to development of a variety of diseases.^40^ In particular, FATP1 upregulation in cancer cells plays a pivotal role in lipid uptake from the microenvironment, contributing to the proliferation and survival of these cells, and its inhibition decreases proliferation and invasiveness of cancer cells.^34^^,^^41^ The scavenger receptor CD36 also participates in the internalization of long chain fatty acids and is highly expressed in tumor, but not normal, tissues.^39^^,^^42^ This elevated expression leads to accumulation of lipids, which contribute to cell proliferation, signal transduction, and fatty acid-induced metastasis, indicating a pro-tumorigenic role for CD36. Furthermore, higher expression of CD36 has been associated with poorer patient prognosis in numerous tumor types.^42^, ^43^, ^44^ The importance of this transporter was also demonstrated in a study showing that the protective effect of oleic acid on SCD1 inhibitor-induced cytotoxicity was prevented by CD36 depletion.^39^

Although low levels of CD36 protein expression were observed in untreated cells, this may be due to culture in medium containing 10% serum, as low expression of CD36 mRNA was previously reported in LNCaP cells cultured in a medium containing 5% serum.^29^ Expression of both CD36 and FATP1 proteins was, however, upregulated after culture in reduced serum concentrations or treatment with C75. This is likely in response to the reduced availability of fatty acids in the medium or the increased requirement for exogenous fatty acids when FASN is inhibited and was also observed in response to AMP-activated kinase activation.^37^ We also observed increased expression of CD36 and FATP1 protein after irradiation, similar to the increase in CD36 and FATP1 mRNA observed in rat liver after irradiation.^45^

It has been suggested that targeting fatty acid uptake and transport may be a promising treatment option for tumors with elevated fatty acid levels, particularly in combination with drugs affecting lipogenesis.^28^^,^^39^ Knock down of CD36 using small interfering or short hairpin RNA, respectively decreased viability and migration of breast cancer cells in vitro^44^ and reduced tumor growth in murine models of glioblastoma and ovarian cancer.^42^^,^^46^ CD36 neutralizing antibodies also inhibited growth of breast cancer cells,^44^ metastasis in mouse models of human oral cancer,^43^ and cancer severity in patient-derived prostate cancer xenografts.^28^ Therefore, we assessed the ability of a CD36 neutralizing antibody to affect viability and clonogenic survival of prostate cancer cells, either as a single agent or in combination with C75 or radiation. We demonstrate here that a CD36 neutralizing antibody reduced cell viability and clonogenicity as a single treatment. Crucially, the antibody also enhanced the effect of C75, similar to the effect observed in human prostate cancer-derived organoids,^28^ further supporting the view that combinations of treatments targeting the synthesis and uptake of fatty acids may have therapeutic potential. We also show, for the first time, that although reducing fatty acid availability or uptake did not enhance radiation-induced cytotoxicity, the effect of radiation in combination with a radiosensitizing FASN inhibitor was enhanced. A similar approach may be possible by targeting FATP1, as suppression of FATP1 was shown to enhance the pro-apoptotic and anti-proliferative effect of FASN silencing in liver cancer cells.^34^

Although Orlistat has been approved as an anti-obesity drug, it has not been approved for cancer therapy as a single agent, and our data indicate that it is unlikely to have potential as a radiosensitizing agent. FASN inhibitors from TVB appear to be safe in humans and reduced lipogenesis in small clinical studies as a single agent and demonstrate some efficacy in patients with cancer. However, no radiosensitizing effect was apparent in this study. C75 had the greatest radiosensitizing effect, and the use of enantiomers may be a means to overcome the potential clinical side effects. Our demonstration that the cancer killing efficacy and radiosensitization of C75 can be further enhanced by combination with inhibitors of fatty acid uptake may provide a mechanism to increase the clinical potential of this drug.

## Conclusions

In summary, we have demonstrated that inhibitors of fatty acid synthase have distinct effects on cytotoxicity of prostate cancer cell lines. It appears that not all FASN inhibitors enhance sensitivity to ionizing radiation at the concentrations and exposure times used here. We also observed that C75-induced cytotoxicity is particularly sensitive to alterations in serum levels and suggest that this was due to availability of exogenous fatty acids. The uptake of fatty acids was also regulated by serum levels, and supplementation of medium with oleic acid increased its uptake and utilization, rescuing cells from C75-induced cytotoxicity. Expression of fatty acid transporter proteins is regulated by fatty acid availability, and blocking CD36-dependent uptake enhanced the sensitivity of prostate cancer cells to inhibition of FASN and a combination of C75 with radiation, indicating that it should be considered as an addition to the therapeutic options for tumors that depend on high levels of lipids for growth.
